# Psoriasis vulgaris presenting as a Koebner isomorphic response

**DOI:** 10.1093/omcr/omab069

**Published:** 2021-08-13

**Authors:** Gautam Srivastava, Govind Srivastava

**Affiliations:** 1 Faculty of Life Sciences and Education, University of South Wales, Pontypridd, UK; 2 Department of Dermatology and Venereology, Skin Institute and School of Dermatology, New Delhi, India

**Keywords:** psoriasis, isomorphic phenomenon, Koebner phenomenon, reverse Koebner phenomenon

A 49-year-old male presented in the outpatient department with an asymptomatic eruption on the lower back of a month’s duration. He had a history of scratch injury 2 months ago while sitting on a public bench, which healed uneventfully. He had no other co-morbidities. On physical examination, erythematous papules with adherent white scales were noted in a linear fashion. Auspitz’s sign was positive and suggested a diagnosis of psoriasis vulgaris (Figure 1). A skin biopsy from a satellite lesion revealed prominent parakeratosis, hypogranulosis, club-shaped rete ridges and papillomatosis. The localization of the psoriasis lesion on the recent injured site pointed towards a Koebner isomorphic phenomenon. The intriguing factor, however, was the lack of history of psoriasis prior to the development of these lesions and their localization only over the injury site. The patient was given a combination of clobetasol and salicylic acid ointment which resolved the lesions in a 2-week period.

**Figure 1:**
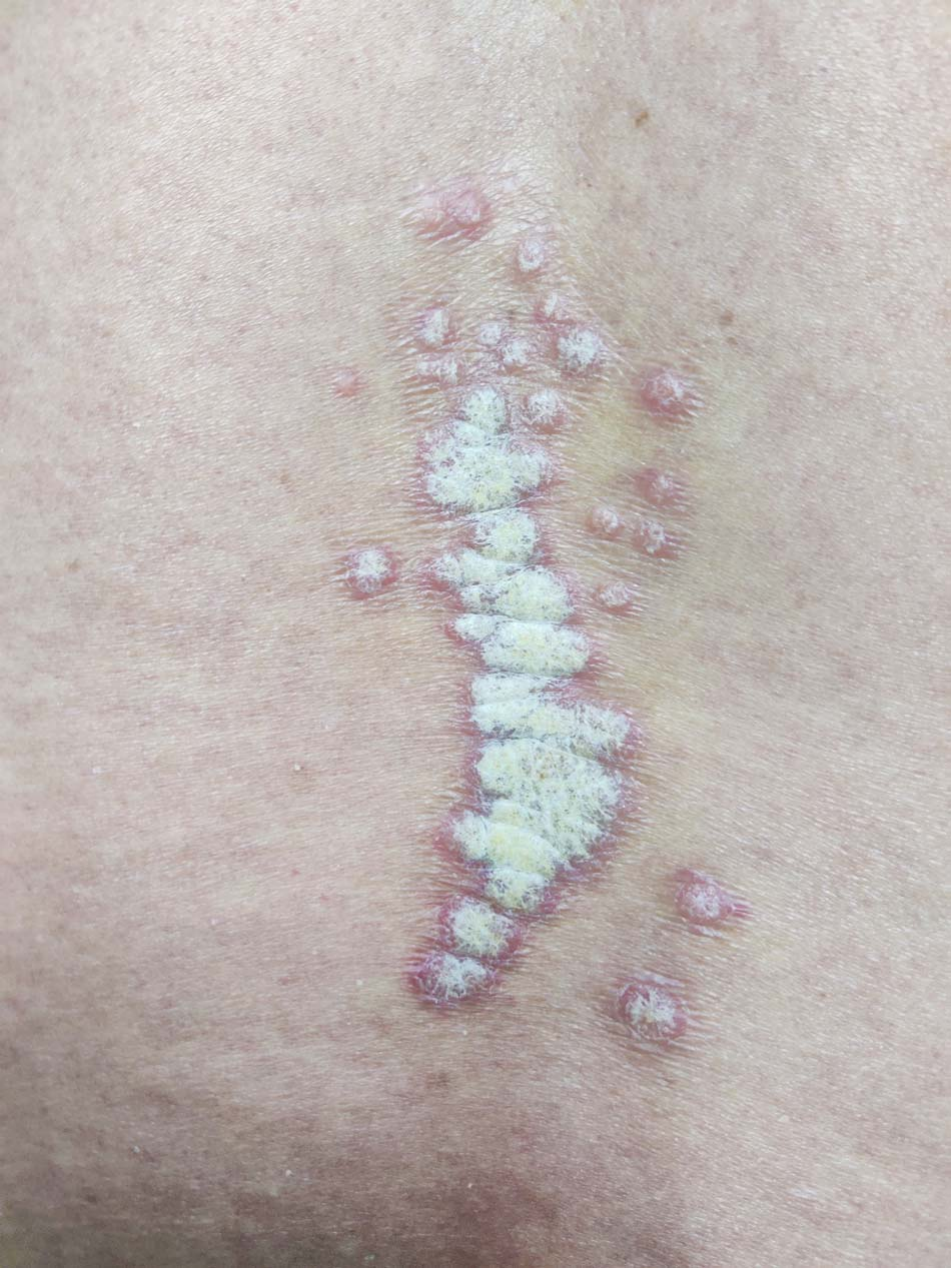
Linear, erythematous and scaly plaque on the lower back overlying the spine which followed a blunt injury.

Heinrich Koebner first described this phenomenon of occurrence of new skin lesions on a previously unaffected area of skin following trauma [1]. This is commonly seen in diseases such as psoriasis, lichen planus and vitiligo. A pseudo-Koebner phenomenon has also been described in molluscum contagiosum and verruca where the infective agent causes the spread of monomorphic lesions in previously unaffected skin [2]. A reverse Koebner phenomenon occurs when the existing skin lesions disappears following trauma [3]. Our case was peculiar in such that psoriasis manifested first at the site of trauma without any previous history.

## CONFLICT OF INTEREST

None declared.

## FUNDING

There was no funding for this publication.

## ETHICAL APPROVAL

Not applicable.

## CONSENT

The authors confirm that written consent for the publication of this case, including images and associated text, has been obtained from the patient.

## GUARANTOR

Dr Govind Srivastava is the guarantor for this publication.
